# Induction of human chronic myeloid leukemia K562 cell apoptosis by virosecurinine and its molecular mechanism

**DOI:** 10.3892/mmr.2014.2531

**Published:** 2014-09-03

**Authors:** GANG ZHANG, MAIDONG LI, SHUWEN HAN, DONGYUN CHEN, YING WANG, WENCAI YE, ZHAONING JI

**Affiliations:** 1 Department of Oncology, Wannan Medical College, Wuhu, Anhui 241001, P.R. China; 2 Department of Medical Oncology, The Cancer Center, Yijishan Hospital of Wannan Medical College, Wuhu, Anhui 241001, P.R. China; 3 Department of Pharmacy, Institute of Traditional Chinese Medicine and Natural Products, Jinan University, Guangzhou, Guangdong 510632, P.R. China

**Keywords:** virosecurinine, apoptosis, mammalian target of rapamycin, SH2 domain-containing inositol-5′-phosphatase 2, phosphatase and tensin homologue, breakpoint cluster region/Abelson, chronic myeloid leukemia, K562 cells

## Abstract

Virosecurinine is a major alkaloid of the plant *Securinega suffruticosa* and has been found to be a potent agent in inducing the differentiation of cancer cells. The present study aimed to investigate the antitumor effects of virosecurinine by inducing the apoptosis of leukemic K562 cells and to examine the underlying mechanisms. K562 cells were treated with different concentrations of virosecurinine (6.25, 12.5, 25, 50, 100 and 200 μmol/l) for 24, 48 and 72 h. The cell counting kit (CCK)-8 method was used to detect the antitumor effect of K562 cells *in vitro*. Flow cytometry was used to observe the apoptotic ratio and analyze the cell cycle following treatment with virosecurinine in K562 cells. Light and electron microscopy was used to identify morphological alterations in the virosecurinine-treated K562 cells. The mRNA levels of mammalian target of rapamycin (mTOR), SH2 domain-containing inositol-5′-phosphatase 2 (SHIP2), phosphatase and tensin homologue (PTEN) and breakpoint cluster region (BCR)/Abelson (ABL) were detected pre and post-virosecurinine treatment using reverse transcription quantitative polymerase chain reaction (RT-qPCR). The generation depression effects of K562 cells cultured *in vitro* were detected using CCK-8 technology, which revealed a dose and time-dependent association. The IC50 was 32.984 μmol/l at 48 h. Flow cytometric analysis indicated that treatment with virosecurinine at concentrations of 6.25, 25 and 50 μmol/l increased the apoptotic rate of the K562 cells and caused G1/S phase arrest. RT-qPCR indicated that virosecurinine upregulated the gene expression of PTEN and downregulated the expression of mTOR, SHIP-2 and BCR/ABL in K562 cells. Virosecurinine inhibited the growth and proliferation of the K562 cell lines and induced apoptosis in K562 cells by affecting the expression of mTOR, SHIP2, BCR/ABL and PTEN.

## Introduction

Chronic myeloid leukemia (CML) is a malignant hematological disease affecting the hematopoietic stem/progenitor cells. CML is characterized by the presence of a constitutively active tyrosine kinase, known as the BCR/ABL oncoprotein, which is produced as a result of a reciprocal translocation between chromosomes 9 and 22 (1). In the United States, 5,000 patients are diagnosed with CML each year (2). However, CML remains one of the most difficult malignant hematological diseases to treat. Chemotherapy is a common therapeutic, however, certain natural anti-tumor medicines, including camptothecin, vinblastine and paclitaxel often cause problems, including adverse reactions and drug resistance. Therefore, the development of an effective natural antitumor drug is required (3).

Securinega alkaloids are a group of natural compounds, which are isolated from the Euphorbiaceae family of plants. Securinine, a major alkaloid found in the leaves of *Securinega suffruticosa,* was initially isolated in 1956 and the structure was determined in 1963 (4). Although securinine, the first alkaloid from this class, was found to be a specific γ-aminobutyric acid receptor antagonist with significant *in vivo* central nervous system activity (5), further examination of this class of compounds led to the identification of molecules exhibiting potent biological properties, including antimalarial (6) and antibiotic properties (7). There are two optical isomers, l-securinine and virosecurinine. Previous pharmacological studies have demonstrated that virosecurinine also possesses antitumor properties (8), improves bone marrow function (9) and induces apoptosis (10). Therefore, there is potential for the clinical use of virosecurinine in the treatment of cancer. Apoptosis is a physiological mechanism for the elimination of cells, which occurs during embryonic development, hormone-induced atrophy and normal cellular homeostasis (11). Since the definition of apoptosis by Kerr (23) in l972, it has been observed that apoptosis is also involved in certain cases of drug-induced tumor cell death (12–13). Numerous currently used anticancer drugs kill particular types of tumor cells via apoptosis.

In the present study, the effect of virosecurinine on the apoptosis of human leukemic K562 cells was investigated to further elucidate the underlying mechanism. In addition, the present study aimed to investigate the efficacy of natural anti-tumor drugs.

## Materials and methods

### Chemicals

**Figure 1:**
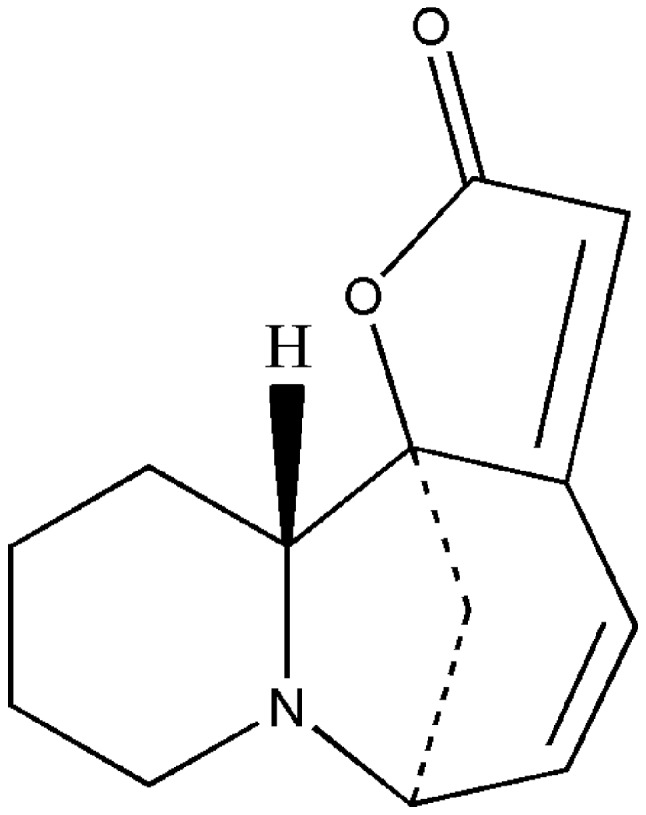
Structure of virosecurinine.

Isolation and extraction of pure virosecurinine (Fig. 1) from *Securinega suffrutico (*Pall.) Rehd was achieved using the ion exchange resin method*.* The pure sample of virosecurinine was provided by the Institute of Traditional Chinese Medicine and Natural Products, Jinan University (Guangzhou, China). The Cell Counting kit-8 (CCK-8) was purchased from KeyGen Biotech Co., Ltd. (Nanjing, China; cat no. KGA317). The cell culture media and solutions were obtained from Gibco-BRL (Carlsbad, CA, USA; cat no. 31800-105).

### Cell culture

The human leukemic K562 cell line was purchased from KeyGen Biotech Co., Ltd. The K562 cells were cultured in RPMI-1640 (Gibco-BRL; cat no. 31800-105) containing 10% fetal bovine serum (FBS; Hangzhou Sijiqing Biological Engineering Materials Co., Ltd., Hangzhou, China; cat no. 120316) and 100 U/ml each of penicillin and streptomycin (KeyGen Biotech Co. Ltd). Cells were grown and maintained at 37°C in a 5% CO_2_ humidified atmosphere.

### Analysis of cell viability

CCK-8 was used to measure cell viability. Exponentially growing K562 cells (100 μl, 5×10^4^ cells/ml) were seeded into 96-well plates (Corning Incorporated, Corning, NY, USA; cat no. 3599). After 24 h, the K562 cells were cultured in RPMI-1640 medium containing 10% FBS and then treated with virosecurinine at concentrations ranging between 6.25 and 200 μmol/l. This was repeated six times at each concentration. The plates were then incubated in a humidified incubator (Sanyo XD-101; Sanyo, Osaka, Japan) at 37°C and 5% CO_2_ for 24, 48 and 72 h. Subsequently, 3 h prior to measuring the absorbance, 10 μl CCK-8 solution was added to each well. The optical density was measured at an absorbance of 450 nm using a microplate reader (ELx800; BioTek Instruments, Inc., Winooski, VT, USA). The inhibitory rate of cellular proliferation was calculated using the following formula: Cellular proliferation inhibitory rate = (1 - mean A450 of experimental group / mean A450 of control group) × 100%.

### Electron microscopy

The K562 cell suspension was seeded at a density of 5×10^5^ cells/well in 6-well plates and was treated with or without virosecurinine at a concentration of 25 μmol/l for 48 h at 37°C. Following incubation, the cells were collected and fixed for 2 h at 4°C in 2.5% ice-cold glutaraldehyde (KeyGen Biotech Co., Ltd.) and then washed three times in 0.1 mol/l phosphate-buffered saline (PBS). The cells were then post-fixed at 4°C in 1% osmium tetroxide (KeyGen Biotech Co., Ltd.) for 2 h and dehydrated through serial dilutions of ethanol (50, 70, 90 and 100% each for 15 min and three times at 100%) prior to being embedded in epoxy resin. The embedded cells were then cut into ultrathin sections (50–60 nm) and stained using uranyl acetate and lead citrate (KeyGen Biotech Co., Ltd.). The sections were viewed using a transmission electron microscope (TEM-1011; Jeol, Tokyo, Japan).

### Analysis of cell apoptosis

To analyze apoptosis, the K562 cells were cultured in 6-well plates (cat no. 3516; Corning Incorporated) with at least 3.0×10^5^ cells/well in medium containing RPMI-1640 (Gibco-BRL, Carslbad, CA, USA), 10% FBS (Sijiqing Biological Engineering Materials, Hangzhou, China) and 100 U/ml penicillin and streptomycin (KeyGen Biotech Co., Ltd.) for 24 h. The cells were then treated with different concentrations of virosecurinine (6.25, 25 and 50 μmol/l) for 48 h. The cells were washed twice with cold PBS (cat no. KGB500; KeyGen Biotech Co., Ltd.), followed by the addition of 5 μl annexin V-fluorescein isothiocyanate (FITC; cat no. KGA105; KeyGen Biotech Co., Ltd.) and propidium iodide (PI; cat no. KGA511; KeyGen Biotech Co., Ltd). After 15 min incubation at room temperature in the dark, the cells were analyzed using flow cytometry. Fluorescence was measured using a FACScan flow cytometer (Becton-Dickinson, Franklin Lakes, NJ, USA) equipped with an argon laser (488 nm). The cell apoptotic rate was calculated using the internal software system of the FACScan (Becton-Dickinson).

### Cell cycle analysis

For cell cycle analysis, the K562 cells were cultured in 6-well plates (at least 3.0×10^5^ cells/well) with medium containing RPMI-1640, 10% FBS and 100 U/ml penicillin and streptomycin for 24 h and then treated with different concentrations of virosecurinine (6.25, 25 and 50 μmol/l) for 48 h. The cells were washed, collected, fixed using 70% ethanol and stored at 4°C overnight. Following that, the cells were treated with Tris-HCl buffer (pH 7.4) containing 1% RNase A (cat. no KGA511; KeyGen Biotech Co., Ltd) and stained using PI (5 mg/ml). Flow cytometry (FACSCalibur; Becton-Dickinson) was used to determine the distribution of cells with different DNA contents. The data were analyzed using multicycle DNA content and cell cycle analysis software (FlowJo, version 7.6.5; KeyGen Biotech Co., Ltd.).

### Reverse transcription quantitative real-time polymerase chain reaction (RT-qPCR)

RT-qPCR was performed to analyze the mRNA levels of mammalian target of rapamycin (mTOR), phosphatase and tensin homologue (PTEN), breakpoint cluster region (BCR)/Abelson (ABL) and SH2 domain-containing inositol-5′-phosphatase 2 (SHIP2) in the K562 cells treated with or without virosecurinine. Total RNA was isolated from the K562 cells using TRIzol reagent (cat no. 15596-026; Invitrogen Life Technologies, Carlsbad, CA, USA) according to the manufacturer’s instructions. First strand cDNA synthesis was performed using the ProSTARt First Strand RT-PCR kit (cat no. PC0002; Fermentas*,* Vilnius, Lithuania) according to the manufacturer’s instructions. Following reverse transcription, 20 μl of the reaction mixture (cat no. EP0702; Fermentas) was used in a qPCR program (cat no. DA7600; Zhongshan Bio-Tech Co., Ltd., Zhongshan, China) comprising 40 cycles consisting of denaturation (15 sec at 95°C), annealing (20 sec at 60°C) and extension (40 sec at 72°C). The 20 μl reaction mixture contained 10 μM each primer, 2 μl 2X QuantiTect SYBR green RT-PCR master mix, 10 μl QuantiTect reverse transcriptase mix and nuclease-free water (cat no. KGDN4500; KeyGen Biotech Co., Ltd) up to 8 μl. Data were analyzed using the 2^−ΔΔCt^ method. The experiment was repeated three times and the efficiency of cDNA synthesis from each sample was estimated using GAPDH-specific primers. The primers used were as follows: mTOR, forward 5′-ATTTGATCAGGTGTGCCAGT-3′ and reverse 5′-GCTTAGGACATGGTTCATGG-3′; GAPDH, forward 5′-TGTTGCCATCAATGACCCCTT-3′ and reverse 5′-CTCCACGACGTACTCAGCG-3′; PTEN, forward 5′-CAAGATGATGTTTGAAACTATTCCAATG-3′ and reverse 5′-CCTTTAGCTGGCAGACCACAA-3′; BCR/ABL, forward 5′-CTCCAGACTGTCCACAGCATTCCG-3′ and reverse 5′-CAGACCCTGAGGCTCAAAGTCAGA-3′ and SHIP2, forward 5′-GAGCACGAGAACCGTATCAGC-3′ and reverse 5′-CCAAATGAGGTGCCATTAAACA-3′.

### Statistical analysis

All data are expressed as the mean ± standard deviation. SPSS software version 18.0 (SPSS, Inc., Chicago, IL, USA) was used for statistical analyses. Differences between the groups were evaluated using the Student-Newman-Keuls test. P<0.05 was considered to indicate a statistically significant difference.

## Results

### Effect of virosecurinine on the proliferation of K562 cell lines

**Figure 2:**
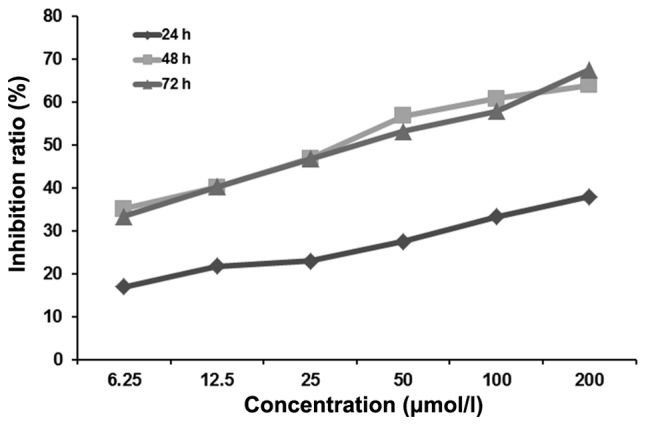
Virosecurinine with dose- and time-dependent inhibition of K562 cells.

In order to understand the mechanism underlying virosecurinine-induced apoptosis in K562 cells, a CCK-8 assay was used to determine the effects of virosecurinine on the proliferation of K562 cells. The K562 cells were treated with virosecurinine at concentrations ranging between 6.25 and 200 μmol/l or with dimethyl sulfoxide (DMSO; KeyGen Biotech Co., Ltd.) alone for 24, 48 and 72 h and the number of viable cells were determined. The CCK-8 assay revealed that virosecurinine markedly inhibited the proliferation of K562 cells in a dose- and time-dependent manner (Fig. 2). The inhibitory rate at 48 h was significantly higher compared with at 24 or 72 h and the IC50 was 32.984 μM/l at 48 h.

### K562 cell ultrastructure

**Figure 3:**
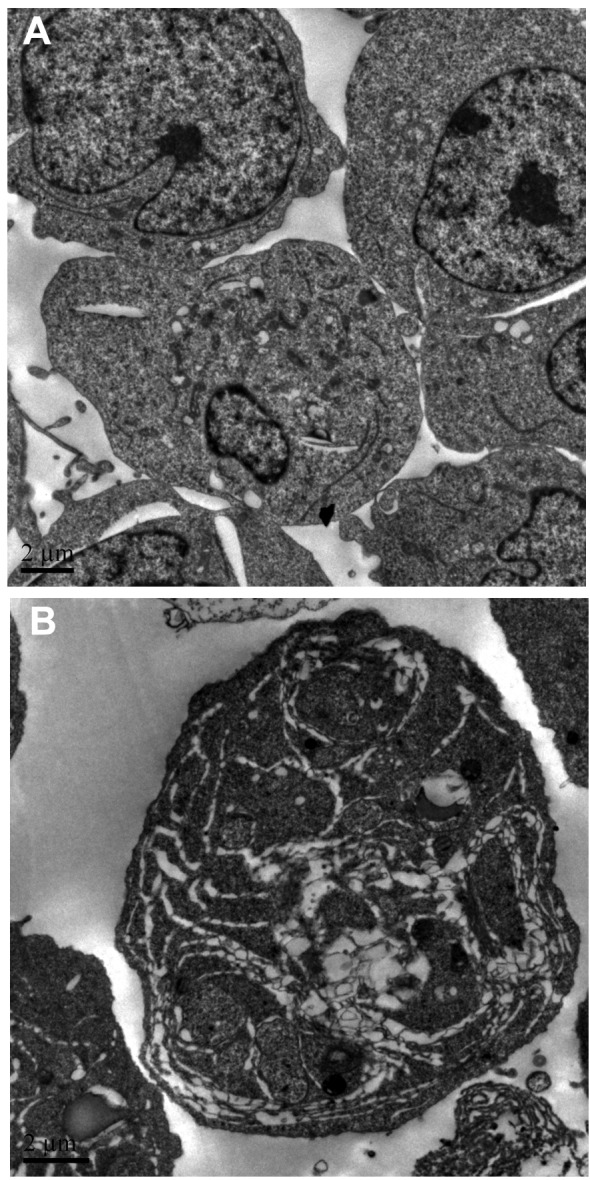
Electron micrographs revealing the ultrastructure of K562 cells treated with virosecurinine (25 μM/l) for 48 h. (A) Control K562 cells treated without virosecurinine for 48 h, revealed few apoptotic bodies. (B) Numerous apoptotic bodies were observed in the virosecurinine-treated K562 cells.

Furthermore, ultra-structural analysis by electron microscopy further confirmed that apoptotic cells were not observed in the DMSO group (Fig. 3A). However, apoptotic bodies were observed in the K562 cells treated with 25 μmol/l virosecurinine for 48 h (Fig. 3B). The assay demonstrated that K562 cells treated with 25 μM/l virosecurinine for 48 h were undergoing apoptosis.

### Flow cytometric analysis of the cell cycle

**Figure 4:**
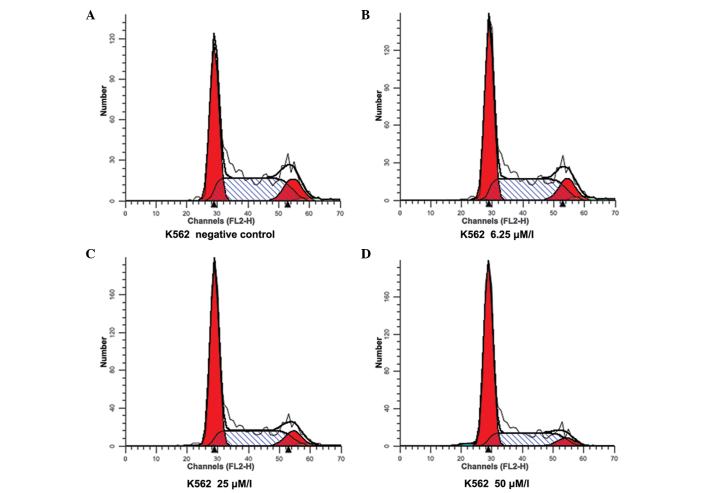
Flow cytometric analysis of the cell cycle treated with virosecurinine. (A) Normal K562 cells. (B-D) Cells treated with 6.25, 25 and 50 μmol/l virosecurinine for 48 h, respectively. The number in the top-right quadrant represents the percentage of autophagic cells.

**Figure 5:**
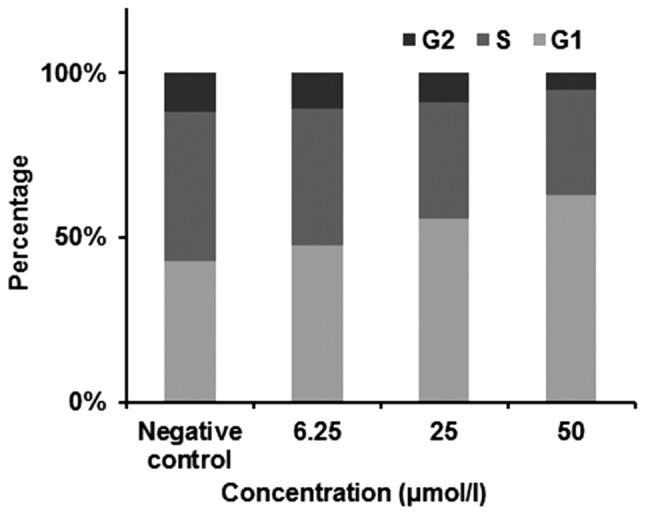
Cell cycle of K562 cells treated with virosecurinine at different concentrations for 48 h and detected by flow cytometry.

To understand the mechanisms of virosecurinine-induced K562 cell apoptosis, the present study analyzed the cell cycle phase distribution. K562 cells were treated with 6.25, 25 and 50 μmol/l of virosecurinine for 48 h and the cell cycle was analyzed using flow cytometry according to the DNA content. The results indicated that virosecurinine arrested the cells at the G1 phase of the cell cycle (Figs. 4 and 5).

### Apoptotic statistical flow cytometric assay

**Figure 6:**
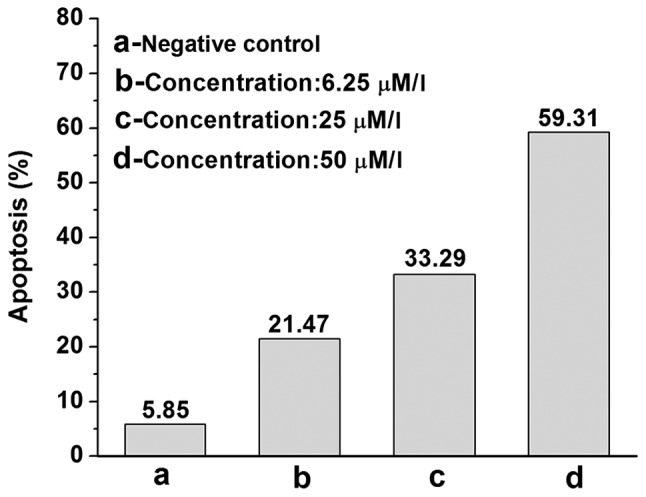
Comparison of K562 cell apoptosis induced by virosecurinine at different concentrations for 48 h and assayed using the annexin V-fluorescein isothiocyanate method.

**Figure 7:**
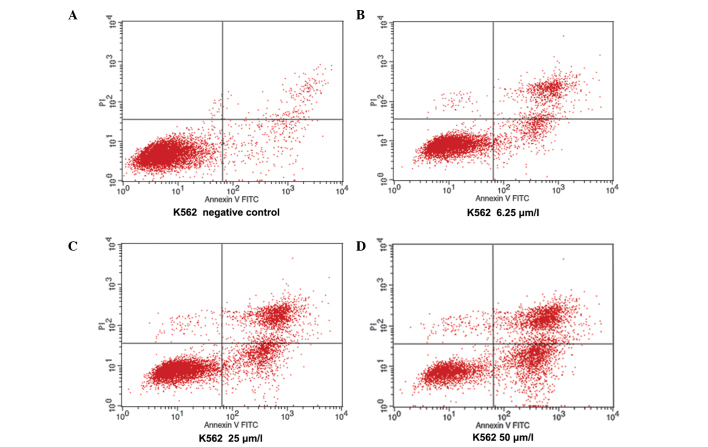
Flow cytometric analysis of apoptotic cells treated with virosecurinine. Apoptotic rates were determined using fluorescence activated cell sorting following virosecurinine treatment in K562 cells. (A) Normal K562 cells. (B-D) K562 cells treated with 6.25, 25 and 50 μmol/l virosecurinine for 48 h, respectively. FITC, fluorescein isothiocyanate.

To further determine whether virosecurinine-treated K562 cells underwent apoptosis, the cells were stained using double-staining with Annexin V and PI. The K562 cells were treated with virosecurinine (6.25, 25 and 50 μmol/l) or without virosecurinine for 48 h. The results demonstrated that virosecurinine increased the percentage of K562 cells undergoing apoptosis in a dose-dependent manner (Fig. 6). The percentage of apoptotic cells following treatment with 6.25, 25 and 50 μmol/l virosecurinine for 48 h was 21.47, 33.29 and 59.31%, respectively (Figs. 6 and 7).

### RT-qPCR analysis of the mRNA expression of mTOR, PTEN, BCR/ABL and SHIP2

**Figure 8:**
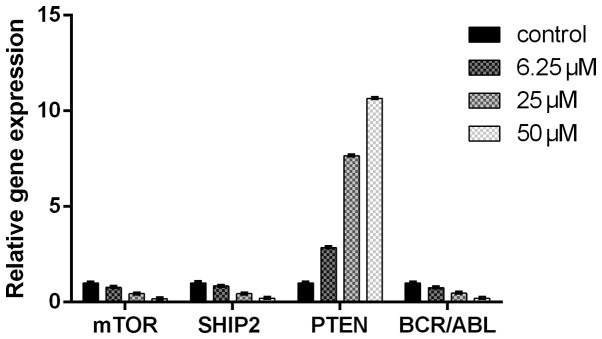
Reverse transcription quantitative polymerase chain reaction analysis of genes in K562 cells treated with virosecurinine at 6.25, 25 and 50 μmol/l for 48 h. mTOR, mammalian target of rapamycin; SHIP2, SH2 domain-containing inositol-5′-phosphatase 2; PTEN, phosphatase and tensin homologue; BCR, breakpoint cluster region; ABL, Abelson.

In order to further understand the molecular mechanism underlying virosecurinine-induced apoptosis, the mRNA levels of mTOR, PTEN, BCR/ABL and SHIP2 mRNA were measured in virosecurinine-deprived and control K562 cells using RT-qPCR analysis. The results indicated that virosecurinine effectively downregulated the expression level of mTOR, SHIP2 and BCR/ABL and upregulated the expression of PTEN (P<0.05; Fig. 8).

## Discussion

Although securinine, a major alkaloid of the plant *Securinega suffruticosa*, has been found to inhibit the growth of MCF-7, SW480 and HL-60 tumor cells, the underlying mechanism remains to be elucidated (4,12). The present study investigated the efficacy of virosecurinine against the human CML cell line K562 and demonstrated that virosecurinine significantly inhibited the proliferation of K562 cells at low concentrations. The IC50 of virosecurinine to K562 cells 48 h after treatment was 32.984 μM/l. This result indicated that virosecurinine markedly inhibited the proliferation of K562 cells in a dose- and time-dependent manner. In addition, the present study demonstrated that virosecurinine induced cell apoptosis, with the observation of apoptotic bodies and the appearance of a typical sub-G1 peak in the K562 cells treated with 25 μmol/l virosecurinine for 48 h. Apoptosis is important in development and the prevention of apoptosis is a hallmark of cancer. The investigation of apoptosis may be important for the development of novel anticancer therapies.

Previous studies from different groups have used mathematical models of apoptosis and applied them to cancer cells (14–16). The data from these studies demonstrate that the inhibitory effect of virosecurinine on the growth of the human CML cell line K562 may be partly due to the induction of apoptosis. mTOR is a serine/threonine protein kinase, a downstream partner in the phosphoinositide 3-kinase (PI3K)/Akt pathway, which regulates protein translation, cell growth and apoptosis (17). The PTEN gene is a novel tumor suppressor gene and the PTEN protein has protein phosphatase and lipid phosphatase dual activity, with activity against phosphatidylinositol 3,4,5-trisphosphate, the major bioactive product of PI3K (18). It has been identified as a candidate tumor suppressor gene based on the presence of gene deletion or inactivating mutations in human brain, breast and prostate cancers as well as tumor cell lines (19–22). BCR-ABL fusion proteins result from the chromosomal translocation t(9;22), which produces the Philadelphia chromosome and ultimately leads to CML (23). The activity of BCR-ABL has several effects on cells, causing an increase in proliferation and a reduction in apoptosis, leading to the malignant growth of groups of hematopoietic stem cells. Inhibition of ABL by imatinib, its tyrosine kinase inhibitor, has markedly improved the prognosis of patients with CML (24). It has been suggested that SHIP2 is involved in type 2 diabetes and in obesity (25), as well as cancer and atherosclerosis (26). Therefore, there has been an interest in developing compounds that selectively target SHIP2. A previous study described the inhibition of the catalytic activity of SHIP2 by specific molecular compounds (27). In addition, cell permeable pan-SHIP1/2 inhibitors have been identified, which have been reported to cause cell death in multiple myeloma (28). The identification of SHIP2 specific compounds suggests that SHIP2 may be a potential target with which to treat a range of diseases and also enables a deeper understanding of the role of SHIP2 in the immune system.

To further investigate the molecular mechanism of virosecurinine, the present study measured the expression levels of four genes linked to apoptosis, mTOR, PTEN, BCR/ABL and SHIP2 in virosecurinine-treated K562 cells. The results demonstrated that virosecurinine upregulated the gene expression of PTEN and downregulated the expression of mTOR, BCR/ABL and SHIP2 in the K562 cells. Therefore, the present study demonstrated for the first time, to the best of our knowledge, that virosecurinine induced apoptosis in K562 cells by altering the expression of mTOR, PTEN, BCR/ABL and SHIP2. These results demonstrated that virosecurinine effectively suppressed the proliferation of the human CML cell line K562 and suggested that growth inhibition may be in part due to virosecurinine-induced apoptosis through the downregulation of mTOR, BCR/ABL and SHIP2 and the upregulation of PTEN.

The results from the present study, suggest that virosecurinine may serve as a potential lead for future drug development in the prevention and treatment of CML.
